# Vitamin D intake and status in immigrant and native Swedish women: a study at a primary health care centre located at 60°N in Sweden

**DOI:** 10.3402/fnr.v57i0.20089

**Published:** 2013-05-20

**Authors:** Åsa Andersson, Anne Björk, Per Kristiansson, Gunnar Johansson

**Affiliations:** 1 Gottsunda Primary Health Care Centre, Uppsala, Sweden; 2 Department of Public Health and Caring Sciences, Family Medicine and Preventive Medicine, Uppsala University, Uppsala, Sweden

**Keywords:** immigrants, vitamin D deficiency, primary health care, food, nutrition requirements

## Abstract

**Background:**

Immigration to Sweden from lower latitude countries has increased in recent years. Studies in the general population in other Nordic countries have demonstrated that these groups are at risk of developing vitamin D deficiency, but studies in primary health care patients are rare.

**Objectives:**

The aim of this study is to examine possible differences in plasma-25(OH)-vitamin D levels and intake of vitamin D between Swedish and immigrant female patients in a primary health care centre located at 60°N, where half of the inhabitants have an immigrant background. Another objective was to estimate what foods contribute with most vitamin D.

**Design:**

Thirty-one female patients from the Middle East and Africa and 30 from Sweden were recruited. P-25(OH)D was measured and intake of vitamin D was estimated with a modified food frequency questionnaire (FFQ).

**Results:**

Vitamin D deficiency (plasma-25(OH)D <25 nmol/L) was common among immigrant women (61%). One immigrant woman and half of the Swedish women had optimal levels (plasma-25(OH)D >50 nmol/L). There was a positive correlation between the intake of vitamin D from food and plasma-25(OH)D. Only three women, all Swedish, reached the recommended intake of vitamin D from food. The immigrant women had lower intake compared to Swedish women (median: 3.1 vs. 5.1 µg/day). The foods that contributed with most vitamin D were fatty fish, fortified milk and margarine. Immigrant women consumed less fortified milk and margarine but more meat. Irrespective of origin, patients with plasma-25(OH)D <25 nmol/L consumed less margarine but more meat.

**Conclusion:**

Vitamin D deficiency was common in the immigrant patients and their intake of vitamin D was lower. This highlights the need to target information about vitamin D to immigrant women in order to decrease the risk for vitamin D deficiency. The FFQ was well adapted to its purpose to estimate intake of vitamin D.

Recent research has shown that vitamin D deficiency is more prevalent than previously assumed and seems to have a correlation with a range of diseases, such as musculoskeletal pain, diabetes, depression, coronary heart disease, cancer, psoriasis and multiple sclerosis (1–4). The discovery of the presence of vitamin D receptors in different tissues has also led to an increased interest in this vitamin.

Vitamin D is synthesized in the skin after sun exposure and can also be obtained through nutritional intake (2). Globally, the vitamin D synthesis in skin is most important, but in sun-deprived regions such as northern countries, the nutritional contribution is more important. The skin synthesizing process is dependent on the latitude, skin type, age, clothing, usage of sun screen and air pollution (5). People who spend little time outdoors and whose food has a low content of vitamin D are at risk of developing vitamin D deficiency.

In food, vitamin D exists in two different forms, ergocalciferol (vitamin D_2_) and cholecalciferol (vitamin D_3_). From a nutritional point of view, the cholecalciferol form is most important and is found in fatty fish, egg yolks, meat products, poultry, as well as in fortified products, such as milk and margarine. The ergocalciferol form is found in some wild mushrooms (6).

Today, in Sweden, 15% of the inhabitants were born in another country. The proportion originating from countries at lower latitudes (the Middle East and Africa) has grown. Another 4% have an immigrant background, meaning that they were born in Sweden but both their parents born in another country (7).

Studies on immigrants from lower latitudes (7°–40°N) living in Denmark and Norway have demonstrated a high prevalence of vitamin D deficiency, with the lowest levels in women (8–10). To date, there are few studies of vitamin D status among immigrant groups in Sweden and studies from primary health care patients in Nordic countries are rare (11, 12). Sääf et al. recently studied pregnant women originating from Somalia and showed extremely low levels of plasma 25-hydroxyvitamin D, or 25(OH)D.

Thus, it is of great importance to further examine vitamin D status and the intake of vitamin D among immigrant groups in Sweden.

Vitamin D status is most often determined by measuring 25-hydroxyvitamin D 25(OH)D in serum or plasma. This form of the vitamin is considered to best reflect the body supply (13). A value for 25(OH)D above 50 nmol/L has been regarded as a safe lower reference limit and values <25 nmol/L as deficient (14).

The aim of this study is to determine whether there are differences in plasma-25(OH)D levels and intake of vitamin D between immigrant and Swedish women attending a primary care health centre in Uppsala and to examine what kind of foods contributed most vitamin D.

## Subjects and methods

This study was performed in a district in Uppsala where half the inhabitants have immigrant backgrounds, with two-thirds of them originating from the Middle East (15). The study was carried out in co-operation between a dietician and a general practitioner. Inclusion criteria were female patients born in the Middle East or Africa aged 18–75. Exclusion criteria were on-going treatment for vitamin D deficiency and current pregnancy. Immigrant women attending the primary health care centre were consecutively, irrespective of reason for the appointment, asked if they would be willing to participate in a study concerning vitamin D. Those who were interested in participating received both oral and written information and signed a written consent form. A reference group consisting of 30 women born in Sweden was recruited in the same way at the centre. Four of the patients who were invited to participate chose not to participate, owing to lack of time and interest.

In the group of immigrant women, 31 women chose to participate. They originated from Iraq (8 – 7 of them from Kurdistan), Turkey (4), Palestine (4), Somalia (4), Iran (3), Syria (3), Eritrea (2), Jordan (1), Bangladesh (1) and Armenia (1). The immigrant women had lived in Sweden between 2 and 26 years, with a median time of 12 years.

The inclusion period was from January to March 2009. The women consulted the general practitioner and the dietician during the same appointment but separately from the recruiting appointment. Professional interpreter was used in six cases, and in one case, the husband was present and translated when needed. The general practitioner collected information on general health, medication, smoking, muscle pain, sun habits and reactions to sun exposure, education and, for the immigrants, how many years they had lived in Sweden. Most of these data will be reported elsewhere.

To estimate the intake of vitamin D from food, a semiquantitative food frequency questionnaire (FFQ) specially designed for this study was used (Appendix) The FFQ was designed by a dietician with good knowledge and experience on food habits in the studied ethnic groups. It consisted of 15 foods and 8 frequencies and aimed at estimating intake during the last 2 to 3 months. The FFQ included foods containing vitamin D_3_ naturally, foods fortified with vitamin D_3_ and corresponding foods not fortified, such as full fat milk. The FFQ also included vitamin supplements (brand and contents). The women were asked about the size of portions, numbers of glasses of milk, tablespoons of margarine and numbers of eggs. We carefully asked about which brands of milk and yoghurt they had consumed, and from the respective tables of contents, the amount of vitamin D was estimated. Concerning meat and fish, we assumed a main course to be 125 g, which is a normal serving in Sweden. We did not use pictures of reference portions. If a woman mentioned that she consumed extra large or extra small sizes of portions, the frequency in the FFQ was changed accordingly. Since the vitamin D content in fatty fish is much higher than in lake lean fish, only fatty fish was included. The feasibility of the FFQ was checked with the patients, and all items in the FFQ were discussed together with each participant during the appointment. The interviews lasted 30–60 minutes. The Swedish national food agency database was used for the calculations of vitamin D intake (16).

Plasma levels of 25(OH)D were measured as well as plasma calcium. Plasma-25(OH)D was analyzed using an automatized immunochemical method (LIAISON^®^ (Diasorin)) at the clinical chemistry laboratory, University Hospital in Uppsala. This laboratory takes part in the DEQAS quality programme since many years. According to the laboratory, their method gives accurate values and usually lies within ± 10% from the mean value in DEQAS. Height and weight were measured and body mass index (BMI) calculated. Vitamin D deficiency was defined as plasma-25(OH)D below 25 nmol/L, insufficiency 25–50 nmol/L and optimal levels >50 nmol/L. The participants were informed about the test results and they received oral and written advice about foods rich in vitamin D. Patients who were vitamin D deficient received supplements of calcium and vitamin D, but that is not further discussed in this paper.

## Statistics

An estimation of power carried out before the study, assuming 60% of vitamin D deficiency in the immigrant group and 20% in the Swedish group, indicated that samples from 60 women would be sufficient to reveal a statistically significant difference with 90% power using a two-sided test of significance and *p*<0.05. Data were analyzed with the SPSS (version 20.0) statistical programme packages. Neither the original data nor the log-transformed were normally distributed. Summary statistics such as medians, percentiles, and proportions were therefore computed using standard non-parametric methods. Differences in proportions were calculated with the chi-square test. Linear regression was used to evaluate the relationship between quantitative parameters. *p*-Values <0.05 were considered as statistical significance.

The study was approved by the local ethics committee at Uppsala University (Local ID number: 2008:359).

## Results

Characteristics of the women by ethnic origin are shown in Table 1. The immigrant women were somewhat younger and had a significantly lower intake of vitamin D from food as well as total intake (from food and supplements), lower education (two of the immigrants had never been to school) and lower plasma-25(OH)D. One third of the immigrant women group were veiled. One of the Swedish women was converted into Islam and wore a burqa. Deficiency levels were common in the immigrant women but uncommon in Swedish women. Overall, optimal levels were rare among the immigrants.

**Table 1:** Demographics of the study population

	Immigrant women	Swedish women	*p*
Number	31	30	
Age (years)	35 (29–49)	42 (30–57)	0.19
BMI (kg/m^2^)	28 (24–32)	26 (21–32)	0.36
Veiled	10	1	0.006
Education			0.001
School <10 years	13	1	
School 10–12 years	3	7	
School >12 years	15	22	
Muscle pain (n)			0.086
25(OH)D <25 nmol/L	16	2	
25(OH)D ≥25 nmol/L	11	15	
P-25(OH)-vitamin D (nmol/L)	22.2 (13.7–28.6)	51.5 (39.6–66.5)	<0.001
<25 nmol/L	19	2	<0.001
25–50 nmol/L	11	12	
>50 nmol/L	1	16	
Intake vitamin D (food) (µg/day)	3.1 (2.0–4.4)	5.1 (3.7–6.4)	
Intake vitamin D (food+suppl.) (µg/day)	3.1 (2.0–4.7)	5.8 (4.0–9.0)	<0.001
P-calcium	2.3 (2.3–2.4)	2.3 (2.3–2.4)	0.55

Median (25–75th percentiles) and numbers are presented.

**Fig. 1:**
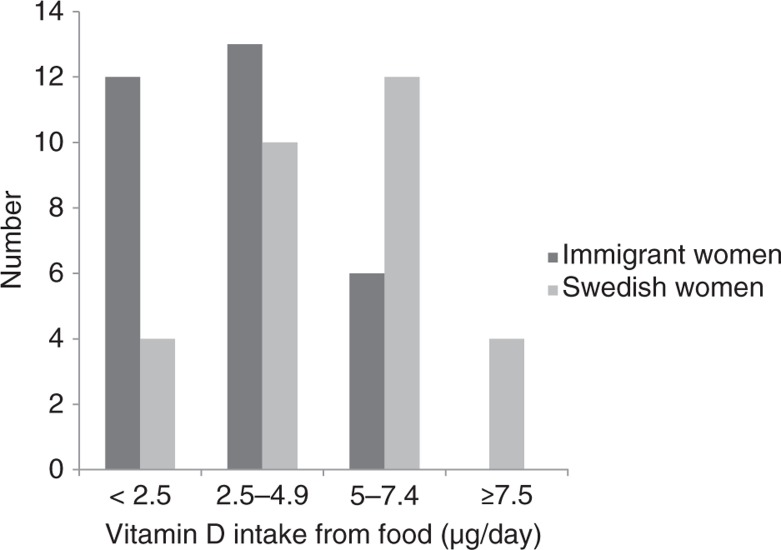
Intake of vitamin D (µg/day) from food in immigrant and Swedish women.

Only three women (Swedish), reached the recommended daily intake for age from food (Fig. 1). Eleven women, two of them immigrant women, took supplements containing vitamin D_3_. Eight Swedish women and two immigrant women reached the recommended level of vitamin D intake when total intake of food and supplements was estimated. One immigrant woman had a high level of plasma-25(OH)D and she had received an injection of vitamin D due to pain while in Iran.

**Fig. 2:**
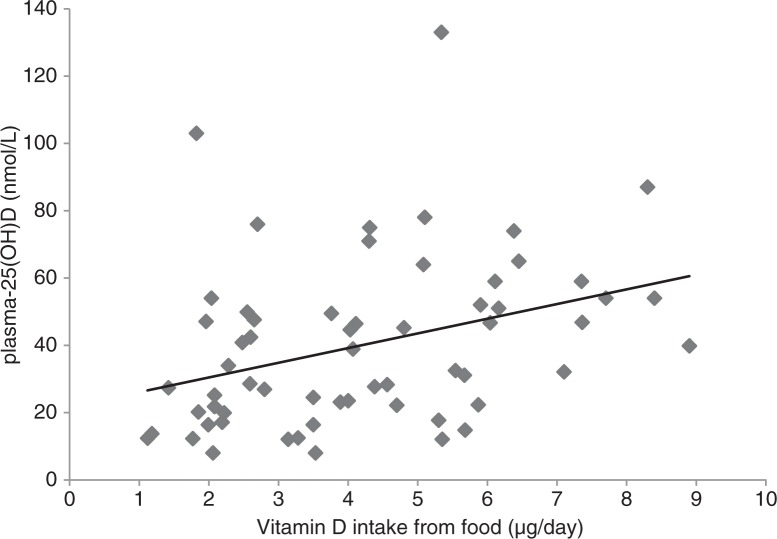
Correlation between vitamin D intake from food and plasma 25(OH)D (nmol/L) in all women. *r*=0.35, *p*=0.006.

There was a positive correlation in the whole group between intake of vitamin D from food and plasma-25(OH)D (Fig. 2). We calculated the intake of vitamin D from different foods and those with a considerable amount are included in Tables 2 and 3. The immigrant and Swedish women obtained their vitamin D from food from different sources (Table 2). The immigrant women had a lower estimated intake of vitamin D from fortified milk and margarine on bread and in cooking than the Swedish reference group. The immigrant women had higher intake of vitamin D from lamb and chicken and the Swedish reference group obtained more vitamin D from pork and ham. The contribution of vitamin D from fatty fish and eggs was similar in the groups.

**Table 2:** Estimated intake of vitamin D (µg/day) from different foods containing considerable amounts of vitamin D in immigrant women (*n*=31) and Swedish women (*n*=30)

	Immigrant women	Swedish women	*p*
Fatty fish	1.41 (0.94–2.02)	1.71 (0.47–2.7)	0.454
Margarine total	0.26 (0.02–0.71)	1.28 (1.25–2.19)	0.011
Fortified low-fat milk	0.05 (0–0.25)	0.25 (0.08–0.76)	0.040
Fortified low-fat yoghurt/sour milk	0 (0–0.08)	0 (0–0.25)	0.273
Eggs	0.12 (0.03–0.28)	0.12 (0.08–028)	0.504
Meat, total intake	0.43 (0.33–0.58)	0.30 (0.26–0.51)	0.029

Median (25–75th percentiles).

**Table 3:** Estimated intake of vitamin D (µg/day) from different foods containing considerable amounts of vitamin D in women with plasma-25(OH)D <25 nmol/L (*n*=21) and ≥25 nmol/L (*n*=40) respective

	<25 nmol/L	≥25 nmol/L	*p*
Fatty fish	1.41 (0.94–2.02)	1.41 (0.47–2.02)	0.763
Margarine, total	0.217 (0.02–1.25)	1.25 (0.52–1.75)	0.001
Fortified low-fat milk	0.05 (0–0.25)	0.25 (0–0.76)	0.038
Fortified low-fat yoghurt/sour milk	0 (0–0.01)	0 (0–0.25)	0.058
Eggs	0.12 (0.03–0.28)	0.12 (0.08–0.28)	0.487
Meat, total intake	0.49 (0.38–0.58)	0.34 (0.27–0.50)	0.021

Median (25–75th percentiles).

Women with plasma-25(OH)D ≥25 nmol/L had a higher intake of vitamin D from fortified milk, fortified margarine on bread and in cooking and pork than women with plasma-25(OH)D <25 nmol/L (Table 3). The women with plasma-25(OH)D <25 nmol/L consumed more lamb, and most of these women were immigrants.

Women with less than 10 years in school had lower plasma-25(OH)D than those with more than 10 years in school (median 19 vs. 45 nmol/L, *p*=0.003).

**Fig. 3:**
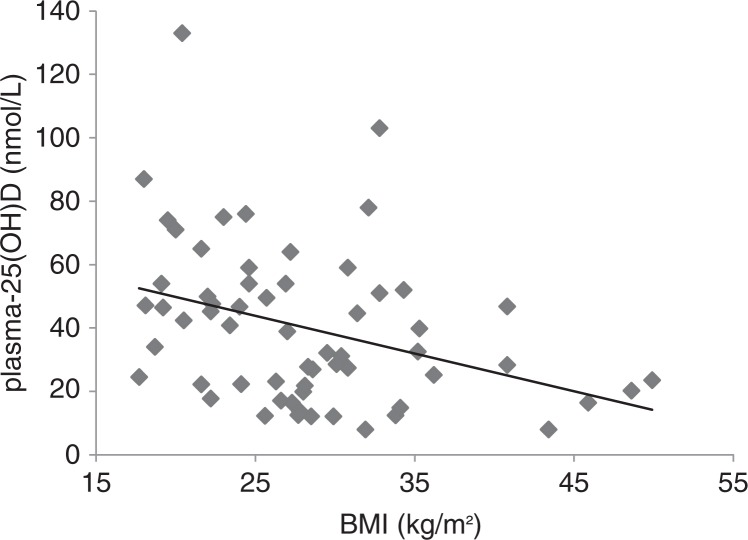
Correlation between BMI (kg/m^2^) and plasma 25(OH)D (nmol/L) in all women. *r*=0.36, *p*=0.005.

Women who were vitamin D deficient had higher BMI and were younger than women with plasma-25(OH)D ≥25 nmol/L. There was a negative correlation between BMI and plasma-25(OH)D in both groups (Fig. 3).

The immigrant women preferred to stay in the shade, while the Swedish reference group chose both sun and shade. There was no difference between plasma-25(OH)D in veiled and non-veiled immigrant women (data not shown).

There was no correlation between years in Sweden and vitamin D status. All women had normal levels of plasma calcium.

## Discussion

We found that in our primary care patients, vitamin D deficiency, defined as plasma-25(OH)D <25 nmol/L, was more common in immigrant women than in Swedish women. However, even though few Swedish women were deficient, many of them had insufficient concentrations of vitamin D. There was a significant correlation between vitamin D intake and plasma-25(OH)D. Immigrant women had lower intake of vitamin D from food and supplements. The main differences in intake of vitamin D from food were that the Swedish women consumed more fortified margarine on bread and in cooking and fortified milk. The other main contributor of vitamin D in food, fatty fish, was consumed in equal amounts in both groups.

Since 1980, the immigration to Sweden from the Middle East and Africa has increased. Within the catchment area of our primary care health centre, half of the population have immigrant backgrounds and of these, two-thirds originate from Middle Eastern countries. Our study population reflects this distribution well. To judge on the basis of clothing habits, skin type and food habits, many of them are at risk of low levels of vitamin D. Immigrants usually maintain most of their traditional food habits in the new country (17). Most of these foods are low in vitamin D. The results from our study show that the immigrant women consumed less margarine on bread and in cooking, presumably because these foods are not common to their traditional food habits.

Fortification of milk, milk products and margarine is one way of increasing intake of vitamin D and has proved to be a strategy to prevent vitamin D deficiency (18). In Sweden and Canada fortification of milk products is compulsory, while it is allowed but not compulsory in other countries (18–21). Present amounts of vitamin D fortification in Sweden are quite low: 0.38 µg/100 g in low-fat milk and low-fat milk products, 7.5–10 µg/100 g margarine and low-fat margarine. Different amounts of fortification in different countries imply difficulties when comparing intake of vitamin D.

Recommended daily intake of vitamin D in Sweden is 10 µg for children aged 0–2, 7.5 µg for people aged 2–60 and 10 µg for people aged >60. Assessment of food intake in Sweden, in a study in which people of age 18–74 years filled in a pre-coded 7-day record book, showed low intakes (average intakes were in the range of 4–6 µg/day) of vitamin D in all ages and for women 4.9 µg/day (22). A new recent national survey (Riksmaten 2010–2011) demonstrated somewhat higher figures for Swedish women, but the response rate was very low (36%) (23). In our study, when using our FFQ, the Swedish women had similar intake as measured in the first survey, while the immigrants had even lower intake of vitamin D.

The United States and Canada recently increased their recommended daily intake of vitamin D, according to a report from the US Institute of Medicine (1). Finland has recently increased their recommended daily intake and prolonged supplementation to children with 7.5 µg vitamin D up to 18 years of age (24). Raising recommendations of daily intake of vitamin D may be important but it is most probably unrealistic to reach these levels by changing food habits. Increased fortification of foods and/ or supplementation may also be necessary.

Another way to increase the daily intake of vitamin D in the population is to recommend prescribed or over the counter (OTC) supplements. The availability of OTC supplements has increased in Sweden during the last years. The dose that has been considered safe for long-term use is 50 µg/day for adults and 25 µg/day for children (25). In 2010, the Institute of Medicine (IOM) raised the upper limit of tolerable intake to100 µg/day for adults (1).

Using an FFQ to estimate vitamin D intake was appropriate in this study. Our FFQ is short and less time-consuming than other methods. It is easy to use for patients whose language skills vary, especially since we discussed each item with the patients. Since some of the women in this study were illiterate, food records were not a possibility. Twenty-four-hour-recall is another option, but in the case of measuring vitamin D it is not a practical alternative since the vitamin exists in high levels in some foods people usually do not consume daily (26). The specially designed FFQ including only foods with relatively high vitamin D content saved time for the respondents and the staff. Baer et al. have shown that FFQ is a useful tool in classifying low-income pregnant American Indian and Caucasian women according to relative dietary intake, and can provide better assessments of usual intake over longer periods of time, such as weeks or months, rather than a single day (27). Taylor et al. showed that data derived from FFQ agree well with those from food record concerning vitamin D (28). They proposed that their FFQ can be effectively used to assess daily intake of calcium and vitamin D. Less robust correlations were observed for calcium intake. Snellman also used a self-administered FFQ consisting of 67 food items to estimate intake of vitamin D in her study of middle-aged and elderly women (29). The difference between our FFQ and the FFQs used by Baer and Taylor was that it was designed to estimate only vitamin D, while they used more items, as they aimed to estimate other nutrients as well. Another difference was that we discussed each item with the patients, while their FFQs were self-administered.

Studies on the general population in Norway, Denmark and Finland have shown low concentrations of serum 25(OH)D for immigrant women from several Asian and African countries (9, 10, 30). Women with higher education in the Norwegian study had higher concentrations of 25(OH)D. This is in accordance with our results, showing higher levels of plasma-25(OH)D in women with >10 years education than in women with <10 years. In the Norwegian study, one in four women reported taking vitamin D supplements. In our study, only 7% of the immigrant women reported supplement intake. In Norway, vitamin D supplement with cod liver oil containing 10 µg vitamin D/5 mL, is common among ethnic Norwegians, which probably also increases the awareness of the importance of vitamin D.

According to the American Institute of Medicine, blood levels of 50 nmol/L are needed for good bone health for practically all individuals. Other experts suggest a level of 75 nmol/L (31). In the recent years, tests for vitamin D have become more widely used, and confusion has increased about how much vitamin D is necessary. Furthermore, the measurements, or cut-off points, for sufficiency and deficiency used by laboratories to report results have not been set based on rigorous scientific studies, and no central authority has determined which cut-off points to use. A single individual may be deemed deficient or sufficient, depending on the laboratory where the blood is tested (1).

Can genetic factors be important for different metabolisms in people originating from different parts of the world? Physiological differences regarding bone health (e.g. gut resistance to actions of 1,25(OH)_2_D and differences in bone turnover) have been reported in African Americans as compared with whites (32). If vitamin D-deficient immigrants from Africa and the Middle East are not protected from skeletal loss owing to some ethnic/racial differences, major health problems could arise in the coming decades (9).

In our study, there was a strong negative correlation between BMI and plasma-25(OH)D. This is in accordance with other studies (33). Studies of women immigrants from Iran and Turkey have shown that the immigrant women had higher BMI and higher degrees of abdominal obesity than Swedish women (34, 35). It may be especially important to consider vitamin D-status when dealing with obese immigrant women from geographical areas such as the Middle East and Africa (36).

In sun-deprived areas such as Sweden, content of vitamin D in food is important to vitamin D status. At latitude 60°N (where Uppsala is situated) cutaneous synthesis of vitamin D is not detectable during the winter season (37). Clothing habits are important and a Norwegian study of vitamin D status in immigrants showed lower levels in veiled women (8). This is in accordance with our findings, although we found plasma-25(OH)D levels <10 nmol/L even in non-veiled women.

One advantage of our study is that we included illiterate women, which was possible as we discussed each item in the FFQ with the patients personally. Illiterate patients are seldom included in studies, because of difficulties with language barriers and the need for interpreters. In spite of the fact that our study is relatively small, it included more patients than earlier Swedish studies investigating vitamin D in immigrant women (12). Another strength was that the dietician had great knowledge of food habits in the studied, ethnic groups, and that the FFQ was constructed from this knowledge and from foods that are available in Sweden.

One possible limitation of the FFQ is that it does not include some foods containing small amounts of vitamin D, for example cakes, biscuits, pancakes, sauces and some lean lake fish. The semiquantitative design of the FFQ might have caused under- or overestimation of the vitamin D intake depending on the sizes of portions. However, studies have shown that extra questions concerning portion sizes are of little importance (38–40). Previous studies have shown great difficulties in measuring food habits in groups with different ethnic backgrounds. There is a need to develop methods with good validity (34). Although we used an interpreter, there was confusion with language in some cases. This might have had an impact on our results.

Another possible limitation of this study may be the choice of method for analyzing plasma-25(OH)D. Many assays for analyzing 25-hydroxyvitamin D are available, and their comparability is uncertain. HPLC (High-pressure liquid chromatography) is sometimes mentioned as a superior method for analyzing vitamin D (41, 42). The methods may differ in the case of vitamin D_2_ or D_3_, which is usually not of interest for monitoring vitamin D status. In our study, we found that two-thirds of the immigrants had deficient levels of plasma-25(OH)D (<25 nmol/L), but if we had used another method, for example, HPLC, probably fewer women would have had deficient levels. However, the difference in plasma-25(OH)D between our two groups would still remain.

The results from this study highlight the need to target information about vitamin D to immigrant women, independent of veil or skin pigmentation, and to detect vitamin D deficiency. Advice about choosing fortified foods and in some cases taking supplements should be considered. Extended education about the importance of vitamin D to midwives and nurses working in child health care could also prevent vitamin D deficiency in future generations. Another option is to increase fortification levels. However, since the immigrant women used less fortified foods, this would require, in the first place, that the foods would be purchased and consumed by those who need them. Another important aspect is to consider a possible correlation between vitamin D status and symptoms attributable to lack of vitamin D.

## Appendix

**Table:** Food frequency questionnaire (FFQ)

How often do you eat?	Portion	Never	<1 time per month	1–2 times per month	2–3 times per month	1 time per week	2–3 times per week	1 time per day	2–3 times per day
Fatty fish	125 g								
Eggs	60 g								
Fortified milk	2 dl								
Milk	2 dl								
Fortified yoghurt	2 dl								
Yoghurt	2 dl								
Cheese	15 g								
Beef	125 g								
Lamb	125 g								
Pork/ham	125 g								
Chicken	125								
Margarine (for bread)	5 g								
Margarine (for cooking)	15 g								
Butter (cooking)	15 g								
Oil	15 g								
Vitamin supplements	1 tablet								
